# Distinct molecular etiologies of male and female hepatocellular carcinoma

**DOI:** 10.1186/s12885-019-6167-2

**Published:** 2019-10-15

**Authors:** Heini M. Natri, Melissa A. Wilson, Kenneth H. Buetow

**Affiliations:** Center for Evolution and Medicine, School of Life Sciences, Arizona State University, Tempe, AZ USA

**Keywords:** Hepatocellular carcinoma, HCC, Gene expression, eQTL, Sex, Sex as a biological variable

## Abstract

**Background:**

Sex-differences in cancer occurrence and mortality are evident across tumor types; men exhibit higher rates of incidence and often poorer responses to treatment. Targeted approaches to the treatment of tumors that account for these sex-differences require the characterization and understanding of the fundamental biological mechanisms that differentiate them. Hepatocellular Carcinoma (HCC) is the second leading cause of cancer death worldwide, with the incidence rapidly rising. HCC exhibits a male-bias in occurrence and mortality, but previous studies have failed to explore the sex-specific dysregulation of gene expression in HCC.

**Methods:**

Here, we characterize the sex-shared and sex-specific regulatory changes in HCC tumors in the TCGA LIHC cohort using combined and sex-stratified differential expression and eQTL analyses.

**Results:**

By using a sex-specific differential expression analysis of tumor and tumor-adjacent samples, we uncovered etiologically relevant genes and pathways differentiating male and female HCC. While both sexes exhibited activation of pathways related to apoptosis and cell cycle, males and females differed in the activation of several signaling pathways, with females showing PPAR pathway enrichment while males showed PI3K, PI3K/AKT, FGFR, EGFR, NGF, GF1R, Rap1, DAP12, and IL-2 signaling pathway enrichment. Using eQTL analyses, we discovered germline variants with differential effects on tumor gene expression between the sexes. 24.3% of the discovered eQTLs exhibit differential effects between the sexes, illustrating the substantial role of sex in modifying the effects of eQTLs in HCC. The genes that showed sex-specific dysregulation in tumors and those that harbored a sex-specific eQTL converge in clinically relevant pathways, suggesting that the molecular etiologies of male and female HCC are partially driven by differential genetic effects on gene expression.

**Conclusions:**

Sex-stratified analyses detect sex-specific molecular etiologies of HCC. Overall, our results provide new insight into the role of inherited genetic regulation of transcription in modulating sex-differences in HCC etiology and provide a framework for future studies on sex-biased cancers.

## Background

Differences in cancer occurrence and mortality between sexes are evident across tumor types; males exhibit higher rates of cancer incidence and often poorer response to treatment, including some forms of chemotherapy and immunotherapy [1, 2]. While differences in risk factors may explain some portion of the sex-bias, the bias remains after appropriate adjustment for these factors [3, 4]. A recent study examining the mutational profiles of tumors from males and females across The Cancer Genome Atlas (TCGA) found sex differences in mutational profiles, calling for the consideration of sex as a biological variable in studies on cancer occurrence, etiology, and treatment [5]. Despite these underlying molecular differences, sex is rarely considered in the development of cancer therapies.

Across tumor types analyzed, the largest sex differences in autosomal mutational profiles were seen in liver hepatocellular carcinoma (HCC), indicating that male and female HCC are etiologically distinct [5]. Furthermore, HCC exhibits sex-bias in occurrence, with a male-to-female incidence ratio between 1.3:1 and 5.5:1 across populations [6, 7]. The sexes also differ in the clinical manifestation of HCC, males exhibiting an earlier onset and more/larger nodules [8]. HCC is the second leading cause of cancer mortality worldwide, accounting for 8.2% of all cancer deaths [2], and the incidence in the US has doubled in the last 3 decades, attributable to increased rates of obesity [7], calling for the development of new interventions and targeted therapies.

Sex-specific gene regulation may partially underlie differences between the sexes in disease prevalence and severity [9, 10]. Previous work observed extensive sex-biased signatures in gene expression in HCC and other sex-biased cancers [11]. However, this study focused solely on comparing male and female tumor samples, without consideration of sex differences in non-diseased and tumor-adjacent tissues. To understand cancer-specific processes, it is necessary to contrast the sex differences in gene expression identified in HCC with those in non-diseases and tumor-adjacent tissues. For the targeted treatment of tumors, it is necessary to understand whether sex differences in cancer reflect unique cancer-specific changes, or are reflective of healthy sex differences that may underlie observed sex-bias in cancer occurrence and disease etiology.

In addition to sex differences in overall gene expression due to the wide effects of sex as a biological variable, genetic variants may alter gene expression in a sex-specific manner. A pan-cancer analysis of the TCGA dataset identified 128 germline variants altering gene expression levels (eQTLs) in HCC [12]. However, this study purposefully controlled for and removed the effect of sex and, to date, a sex-specific eQTL analysis in HCC has not been performed. Sex-stratified analyses can reveal sex-biased genetic effects on gene expression that may be obscured in a joint analysis of both sexes - e.g. cases where the regulatory variant has a zero or very small effect in one sex, or the eQTL exhibits an opposite effect direction in the two sexes [13]. eQTLs that are discovered in one sex but not in the whole sample analysis are likely to affect gene expression in a sex-dependent manner, and while a combined analysis of both sexes achieves a greater statistical power to detect sex-shared effects, it dilutes the signal of sex-dependent effects [14].

Targeted approaches to the treatment of male and female HCC require the characterization and understanding of the fundamental biological mechanisms that differentiate them. Here, we analyzed data from TCGA and The Genotype-Tissue Expression project (GTEx) to examine the sex-specific patterns of gene expression and regulation in HCC. Here, we have contrasted the sex-biased patterns of gene expression in HCC tumors with healthy and tumor-adjacent liver tissues, allowing us to detect sex differences in gene expression shared between and specific to the different tissues. We show that male and female HCC exhibit differences in the dysregulation of genes and germline genetic regulation of tumor gene expression. Importantly, these orthogonal approaches identify genes that converge in shared pathways, indicating sex-specific etiology in HCC. The results presented here have implications for the development of targeted therapies for male and female HCC.

## Methods

### Data

GTEx (release V6p) whole transcriptome (RNAseq) data (dbGaP accession #8834) were downloaded from dbGaP. TCGA LIHC Affymetrix Human Omni 6 array genotype data, whole exome sequencing (WES), and RNAseq data (dbGaP accession #11368) were downloaded from NCI Genomic Data Commons [15]. In total, RNAseq data from 91 male and 45 female GTEx donors, germline genotypes and tumor RNAseq data from 248 male and 119 female TCGA LIHC donors, as well as paired tumor and tumor-adjacent samples from 28 male and 22 female TCGA LIHC donors were utilized in this study. FASTQ read files were extracted from the TCGA LIHC WES BAM files using the *strip_reads* function of *XYAlign* [16]. We used *FastQC* [17] to assess the WES and RNAseq FASTQ quality. Reads were trimmed using *TRIMMOMATIC IlluminaClip* [18], with the following parameters: seed mismatches 2, palindrome clip threshold 30, simple clip threshold 10, leading quality value 3, trailing quality value 3, sliding window size 4, minimum window quality 30 and minimum read length of 50.

### Read mapping and read count quantification

Sequence homology between the X and Y chromosomes may cause the mismapping of short sequencing reads derived from the sex chromosomes and affect downstream analyses [16]. To overcome this, reads were mapped to custom sex-specific reference genomes using *HISAT2* [19]. Female samples were mapped to the human reference genome GRCh38 with the Y-chromosome hard-masked. Male samples were mapped to the human reference genome with Y-chromosomal pseudoautosomal regions hard-masked. Gene-level counts from RNAseq were quantified using *Subread featureCounts* [20]. Reads overlapping features (genes or RNA families with conserved secondary structures) were counted for each feature.

### Germline variant calling and filtering

BAM files were processed according to Broad Institute *GATK* (*Genome Analysis Toolkit*) best practices [21–23]: Read groups were added with *Picard Toolkit*’s *AddOrReplaceReadGroups* and optical duplicates marked with *Picard Toolkit*’s *MarkDuplicates* (v.2.18.1, http://broadinstitute.github.io/picard/). Base quality scores were recalibrated with *GATK* (v.4.0.3.0) *BaseRecalibrator.* Germline genotypes were called from whole blood Whole Exome Sequence samples from 248 male and 119 female HCC cases using the scatter-gather method with *GATK HaplotypeCaller* and *GenotypeGVCFs* [21]. Affymetrix 6.0 array genotypes were lifted to GRCh38 using the UCSC *LiftOver* tool [24] and converted to VCF. Filters were applied to retain variants with a minimum quality score > 30, minor allele frequency > 10%, minor allele count > 10, and no call rate < 10% across all samples.

### Clinical characteristics and cellular content of tumor samples

Confounding effects, e.g. differences in clinical and pathological characteristics or cell-type composition of the sequenced samples, may contribute to the observed effect modification when utilizing stratified analyses. We examined the differences in the clinical characteristics between males and females in the TCGA LIHC cohort. We used a *t*-test to test for the equality of means in patient age and cell-type proportions, and Fisher’s exact test to test to detect differences in risk factors and pathological classifications (Additional file 3: Tables S1 and S2).

### Filtering of gene expression data

FPKM (Fragments Per Kilobase of transcript per Million mapped reads) expression values for each gene were obtained using *EdgeR* [25]. Each expression dataset was filtered to retain genes with mean FPKM≥0.5 and read count of ≥6 in at least 10 samples across all samples under investigation. In the comparative analysis of differentially expressed genes (DEGs) between the tumor vs. tumor-adjacent samples in males, females, and both sexes, genes that reached the previously described expression thresholds in at least one tissue in at least one sex were retained. This assures that the differences in DEGs detected in the sex-specific and combined analyses are not due to filtering.

### Differential expression analysis

For differential expression (DE) analysis, filtered, untransformed read count data were quantile normalized and logCPM transformed with *voom* [26]. From the TCGA LIHC dataset, paired tumor and tumor-adjacent samples were available for 22 females and 28 males. From the GTEx liver dataset, 91 male and 45 female samples were used in the DE analysis. A multi-factor design with sex and tissue type as predictor variables were used to fit the linear model. *duplicateCorrelation* function was used to calculate the correlation between measurements made between tumor and tumor-adjacent samples on the same subject, and this inter-subject correlation was accounted for in the linear modeling. As the paired tumor samples differed significantly between the sexes in terms of race, tumor grade, and HBV status, (Additional file 3: Tables S1 and S2), these parameters were included in the linear models as covariates. Due to missing values in the covariate data, the final numbers of sample pairs used in the analyses were 18 females and 26 males.

DEGs between comparisons were identified using the *limma*/*voom* pipeline [26] by computing empirical Bayes statistics with *eBayes*. An FDR-adjusted *p*-value threshold of 0.01 and an absolute log_2_ fold-change (FC) threshold of 2 were used to select significant DEGs.

To reliably detect genes that are expressed in a sex-biased way in HCC but not in non-diseased liver or in tumor-adjacent tissue, we examined genes that were DE in the male vs. female tumor comparison using the previously described significance thresholds, but not in the male vs. female comparisons of normal or tumor-adjacent samples with a relaxed significance threshold of FDR-adjusted *p*-value ≤0.1 and absolute log_2_(FC) ≥ 0.

To detect genes that are dysregulated in tumors compared to matched tumor-adjacent samples in each sex, we identified DEGs in the tumor vs. tumor-adjacent comparison of males, females, and in the whole sample. DEGs that were identified in one sex but not in the other or in the combined analysis of both sexes were considered sex-specific. DEGs identified in the combined analysis were considered sex-shared. This approach allows the identification of high-confidence sex-specific events that are a result of the underlying biological differences as opposed to sampling or statistical power. ANOVA and Kruskal-Wallis tests were used to test for equality of fold changes of sex-shared and sex-specific DEGs across male, female, and all samples.

### Overrepresentation of biological functions and canonical pathways

We further analyzed the sex-shared and sex-specific tumor vs. tumor-adjacent DEGs as well as the sex-specific eQTL target genes (eGenes) to identify sex-shared and sex-specific pathways driving HCC etiology. We used the *NetworkAnalyst* webtool [27], which utilizes a hypergeometric test to compute *p*-values for the overrepresentation of genes in regards to GO terms and KEGG and Reactome pathways. An FDR-adjusted *p*-value threshold of 0.01 was used to select significantly overrepresented GO terms and canonical pathways.

### Accounting for confounding effects and population structure

Gene expression values are affected by genetic, environmental, and technical factors, many of which may be unknown or unmeasured. Technical confounding factors introduce sources of variance that may greatly reduce the statistical power of association studies, and even cause false signals [28]. Thus, it is necessary to account for known and unknown technical confounders. This is often achieved by detecting a set of latent confounding factors with methods such as principal component analysis (PCA) or Probabilistic Estimation of Expression Residuals (PEER) [29]. These surrogate variables are then used as covariates in downstream analyses. We derived 10 PEER factors from the filtered tumor gene expression data and used the weights of these factors as covariates in the eQTL analysis. We used the R package *SNPRelate* [30] to perform PCA on the germline genotype data. We accounted for population structure by applying the first three genotype PCs as covariates in the eQTL analysis.

### eQTL analysis

We used eQTL analyses to detect germline genetic effects on tumor gene expression. Similar to the DE analysis, we utilized combined and sex-stratified analyses to detect sex-shared and sex-specific effects. Germline genotypes and tumor gene expression data from 248 male and 119 female donors in the TCGA LIHC cohort were used in the eQTL analysis. Filtered count data was normalized by fitting the FPKM values of each gene and sample to the quantiles of the normal distribution. To account for technical confounders and population structure, 10 de novo PEER factors and three genotype principal components were used as covariates. *Cis*-acting (proximal) eQTLs were detected by linear regression as implemented in *QTLtools* v.1.1 [31]. Variants within 1 Mb of the gene under investigation were considered for testing. We used the permutation pass with 10,000 permutations to get adjusted *p*-values for associations between the gene expression levels and the top-variants in *cis*: first, permutations are used to derive a nominal *p*-value threshold per gene that reflects the number of independent tests per *cis*-window. Then, *QTLtools* uses a forward-backward stepwise regression to determine the best candidate variant per signal [31]. FDR-adjusted *p*-values were calculated to correct for multiple phenotypes tested, and an adjusted *p*-value threshold of 0.01 was used to select significant associations. To allow the comparison of effect sizes of sex-specific and sex-shared eQTLs across the sexes, the effects of each variant located within the 1 Mb *cis*-window were obtained using the *QTLtools* nominal pass.

Similarly to the tumor vs. tumor-adjacent DEGs, eQTLs that were detected in one sex but not in the other or in the combined analysis were considered sex-specific, while eQTLs detected in the combined analysis were considered sex-shared. ANOVA and Kruskal-Wallis tests were used to test for equality of effect sizes of sex-shared and sex-specific eQTLs across male, female, and all samples.

### Estimating statistical power in the eQTL analysis

We used the R package *powereQTL* [32] to estimate the effect of the sample size to the statistical power to detect eQTLs in the combined analysis of both sexes and in the sex-specific analyses (Additional file 2: Figure S2).

### Genomic annotations of eQTLs

We used the R package *Annotatr* to annotate the genomic locations of eQTLs [33]. Variant sites were annotated for promoters, 5’UTRs, exons, introns, 3’UTRs, CpGs (CpG islands, CpG shores, CpG shelves), and putative regulatory regions based on ChromHMM [34] annotations.

## Results

### Sex-specific patterns of gene expression in HCC

**Fig. 1:**
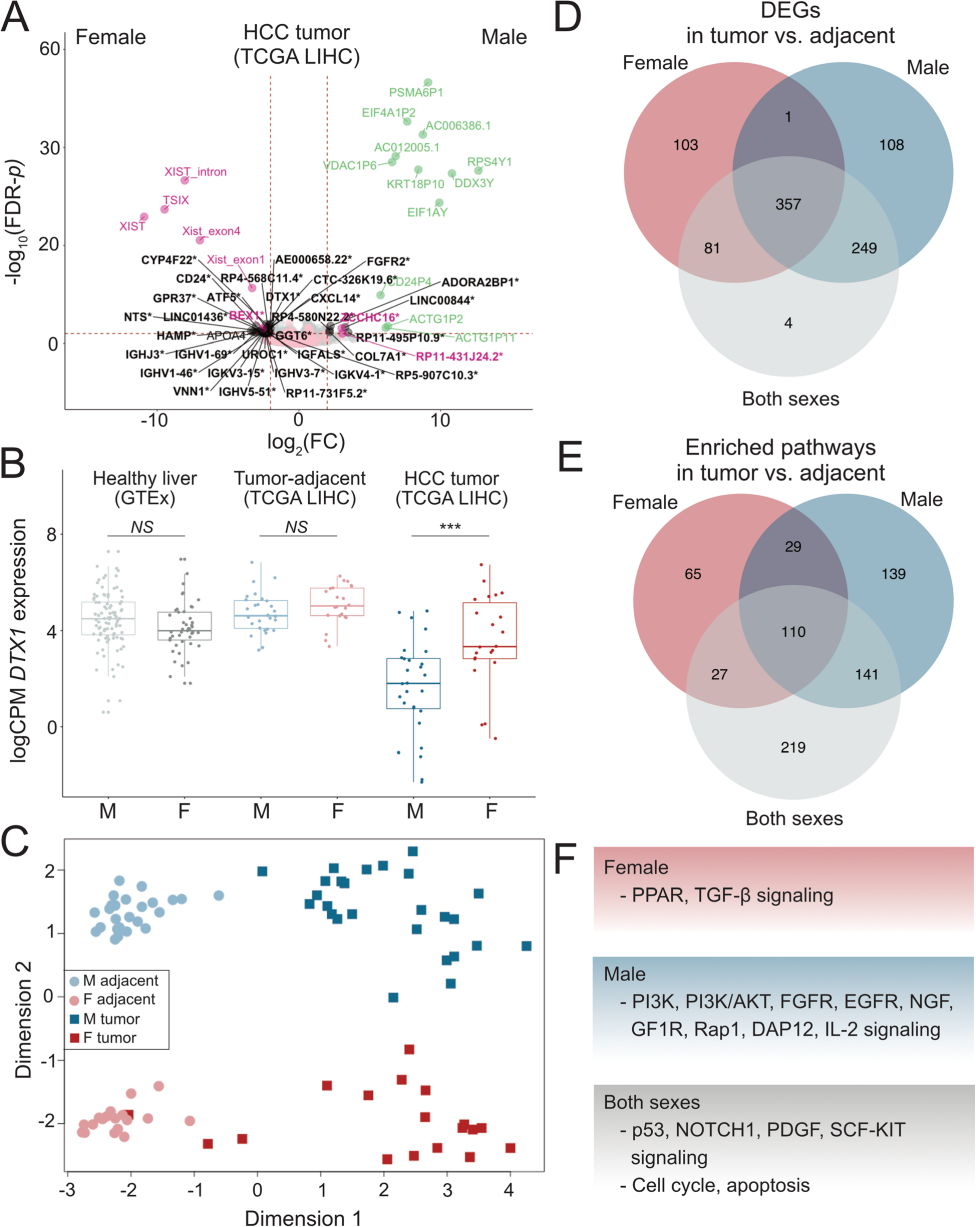
Patterns of gene expression and molecular etiologies of male and female HCC. **a** Sex-biased gene expression in HCC. A volcano plot of DEGs between male (*N* = 26) and female (*N* = 18) HCC tumor samples. X-linked genes are indicated in pink, Y-linked in green, and autosomal in black. Significant genes were selected based on an FDR-adjusted *p*-value threshold of 0.01 and absolute log_2_(FC) threshold of 2. Multiple transcripts of the long non-coding RNA *XIST* are independently expressed. Genes that were not expressed in a sex-biased way in healthy liver (GTEx) or in the tumor-adjacent tissues are indicated with an asterisk. **b** An example of a gene exhibiting a sex-bias in HCC but not in healthy liver or tumor-adjacent tissues. *DTX1* expression in log(CPM) is shown for male and female samples in each tissue. **c** A multi-dimensional scaling plot of the paired TCGA LIHC tumor and tumor-adjacent samples of each sex. Euclidean distances between samples were calculated based on 100 genes with the largest standard deviations between samples. Tissue type (dimension 1) and sex (dimension 2) drive the overall patterns of gene expression in HCC. **d** Venn-diagram of the overlap of DEGs in the sex-specific and combined analyses of matched tumor and tumor-adjacent samples. Substantially more DEGs were identified in the sex-specific analyses. **e** Sex-specific and sex-shared DEGs were analyzed for the overrepresentation of functional pathways. Sex-specific patterns of pathway enrichment point to differential processes driving the etiology of male and female HCC. **f** Examples of sex-specific and sex-shared pathways

We identified sex-differences in gene expression in non-diseased liver (GTEx; 21 sex-biased genes with an FDR-adjusted *p*-value ≤0.01 and an absolute log_2_ (FC)≥ 2), tumor-adjacent tissue (TCGA LIHC; 21 genes), and HCC (TCGA LIHC; 53 genes) to characterize the shared and unique sex differences that may drive the observed sex-biases in HCC occurrence and etiology (Fig. 1, Additional file 3: Tables S3–S5). X-linked *XIST* and Y-linked genes were expressed in a sex-biased way across all tissues. While sex-biased gene expression in non-diseased and tumor-adjacent tissues may contribute to the sex differences in cancer occurrence, sex-biased expression in tumors is suggestive of distinct molecular etiologies of male and female HCC. We identified 34 genes that show sex differences in expression in HCC, but not in tumor-adjacent tissue or non-diseased liver, even with a relaxed significance threshold (Fig. 1a). Notably, Notch-regulating *DTX1* (Fig. 1b) and signal transducer *CD24* were downregulated in male HCC.

**Fig. 2:**
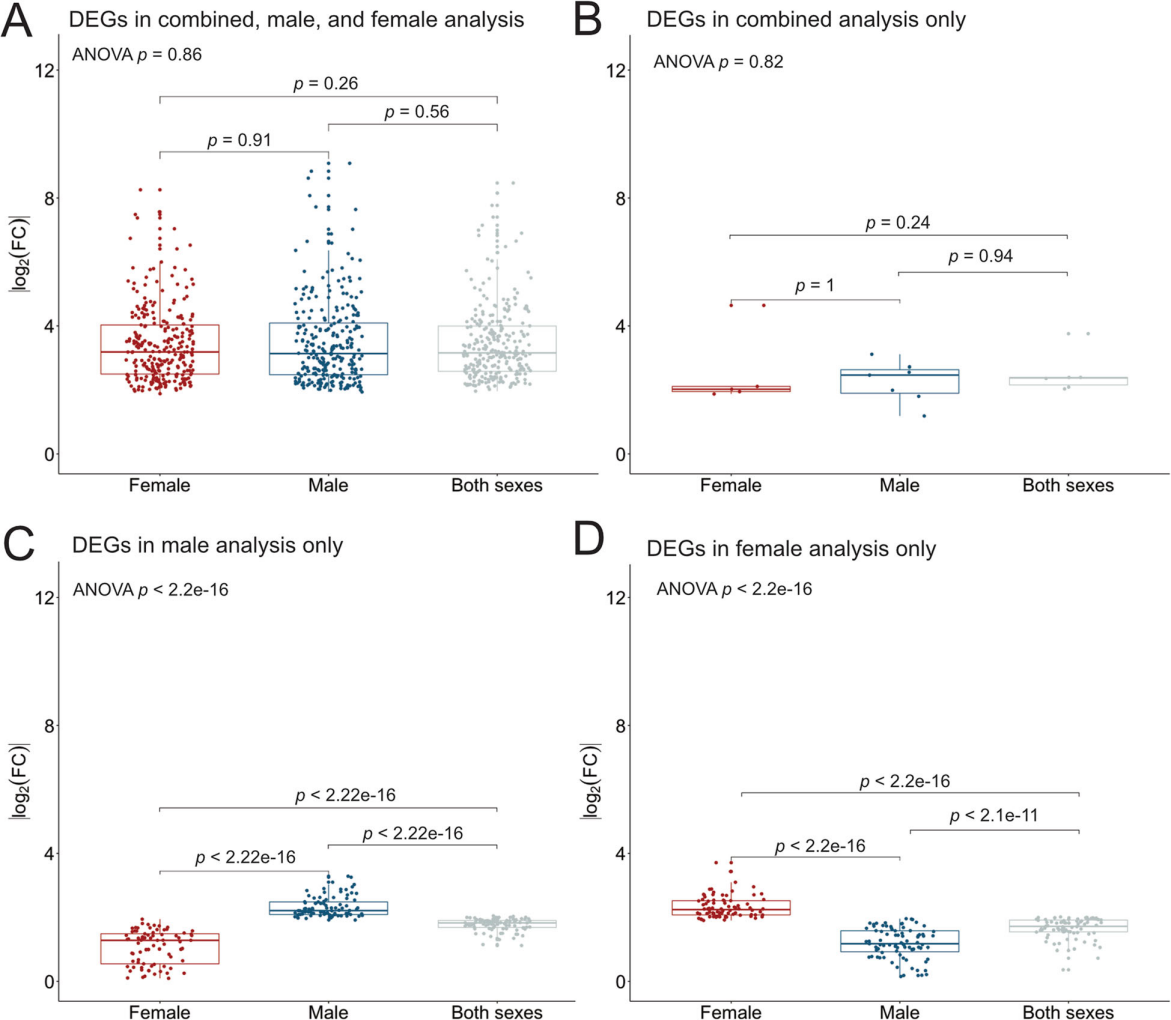
Absolute log_2_-fold changes of DEGs detected from tumor vs. tumor-adjacent comparisons in the combined analysis of both sexes, male, and female analysis (**a**), in the combined analysis only (**b**), in the male analysis only (**c**), and in the female analysis only (**d**). Absolute log_2_-fold changes are given for female samples, male samples, and across all samples. Global *p*-values for ANOVA are shown for each DEG type. Adjusted *p*-values based on Kruskal-Wallis tests are shown for each pairwise comparison

To further examine the sex-shared and sex-specific mechanisms driving HCC etiology, we detected DEGs between tumor and tumor-adjacent samples in males and females, as well as in the combined analysis of both sexes. Dimensionality reduction of gene expression data shows that variation among the tumor and tumor-adjacent samples is driven by tissue type and sex (Fig. 1c). When inspecting the tumor samples only, the first dimension is largely driven by sex (Additional file 1: Figure S1). In the combined analysis of male and female samples, we detected 691 tumor vs. tumor-adjacent DEGs (Additional file 3: Table S6). In male- and female-specific analyses, we detected 715 and 542 tumor vs. tumor-adjacent DEGs, respectively (Additional file 3: Tables S7 and S8). Out of the total of 903 unique DEGs, 76.5% were shared between the sexes. We identified 103 female-specific and 108 male-specific tumor vs. tumor-adjacent DEGs. Notably, substantially more DEGs were detected in sex-specific analyses than in the unstratified analysis (Fig. 1d). Specifically, DEGs that showed different magnitudes in fold change between the sexes (based on ANOVA/Kruskal-Wallis tests) were detected in the sex-specific analyses (Fig. 2c, d), while DEGs with similar fold changes across all comparisons were detected in the combined analysis as well as the sex-specific analyses (Fig. 2a). Sex-shared DEGs that were only detected in the combined analysis, and not in the sex-specific analyses, showed a large variance in expression and, due to limited statistical power, were not detected as statistically significant DEGs in sex-specific analyses (Fig. 2b). Tumor-infiltrating immune cells may produce spurious signals in DE analyses, which is evident from the detection of various immunoglobulin genes in tumor vs. tumor-adjacent comparisons (Additional file 3: Tables S6–8). However, male and female samples did not significantly differ in terms of cellular content (Additional file 3: Table S2), and thus such spurious signals are unlikely to affect male-female comparisons. The observed differences in gene expression are thus likely to reflect actual sex differences rather than confounding differences in sample characteristics or composition.

To put these results in a broader context, we analyzed the male- and female-specific DEGs (tumor vs. tumor-adjacent) for the overrepresentation of functional pathways. We found that the sex-shared and sex-specific DEGs were enriched in pathways relevant to oncogenesis and cancer progression (Additional file 3: Tables S9–S11). We identified pathways that were overrepresented in only one of the sexes but not in the other or in the combined analysis of both sexes, indicating that male and female HCC are partially driven by different mechanisms and processes (Fig. 1e-f).

### Differential *cis*-eQTL effects in male and female HCC

**Fig. 3:**
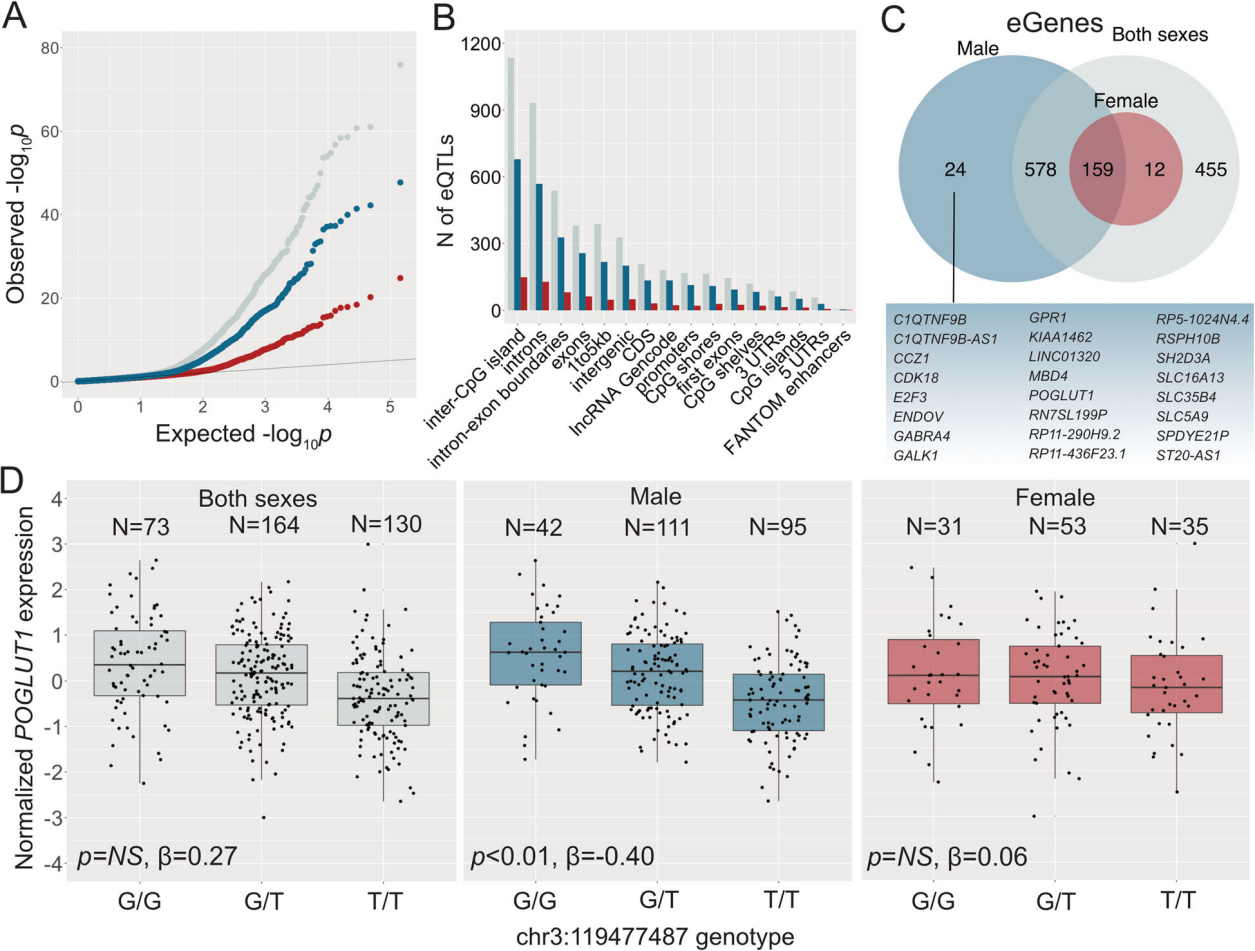
Sex-specific genetic effects on tumor gene expression in HCC. **a** QQ-plot of eQTL associations in the combined analysis of both sexes (grey), male-specific analysis (blue), and female-specific analysis (red). **b** Genomic annotations of eQTLs in the combined analysis of both sexes, male-specific analysis, and female-specific analysis. **c** Overlap of eGenes detected in combined and sex-specific analyses. **d** An example of a male-specific eQTL. *POGLUT1* expression in tumors is modulated by a germline variant in *cis* in male HCC, but not in female HCC nor in the combined analysis of both sexes, indicating effect modification by sex. Numbers of individuals with each genotype, adjusted significance, and effect size (β) are given for each model

**Fig. 4:**
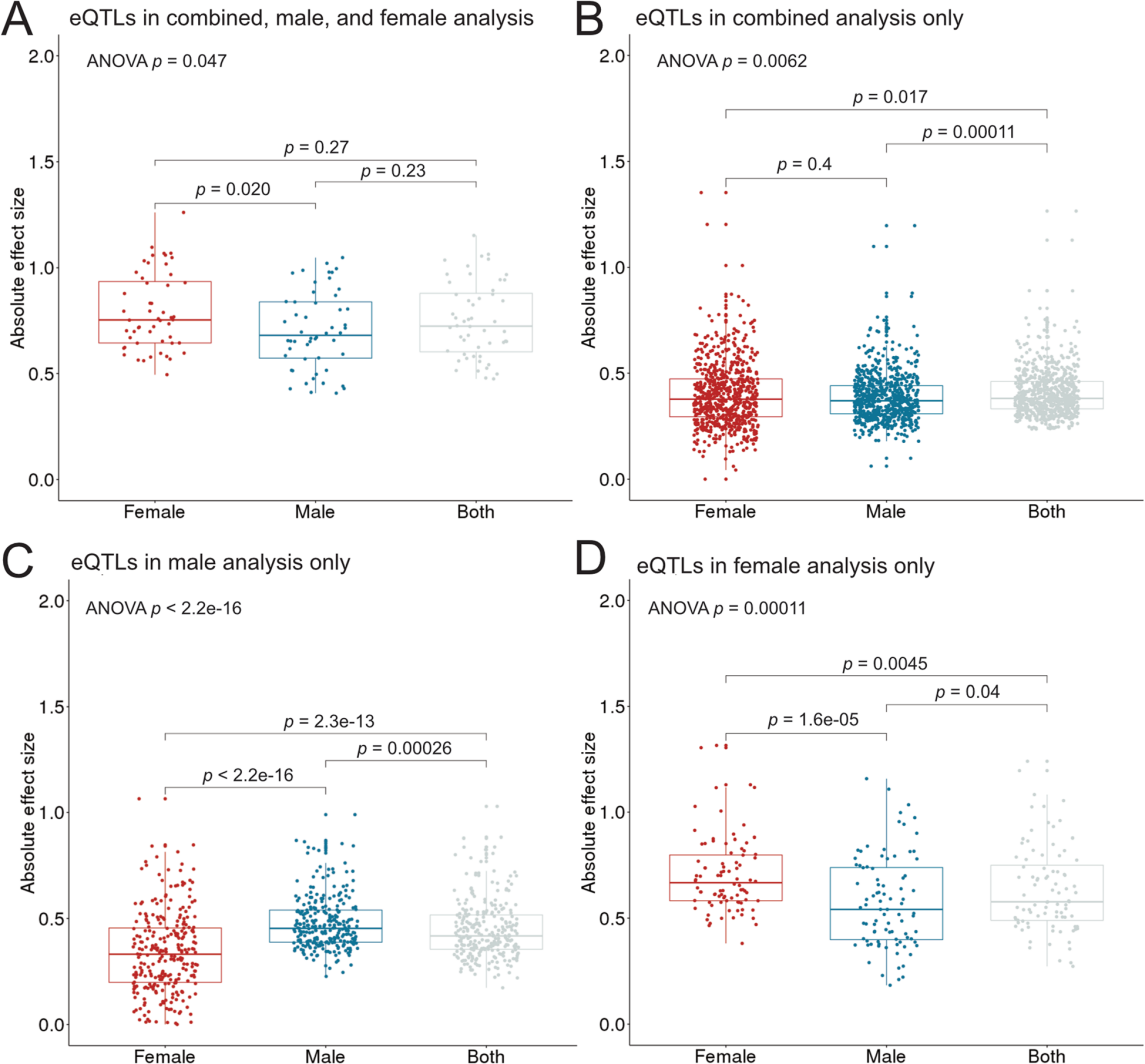
Absolute effect sizes of sex-shared and sex-specific eQTLs in males, females, and the whole study sample. Due to the larger sample size, sex-shared low-effect eQTLs are only detected as significant in the combined analysis (**a**). Sex-shared large effect eQTLs are detected in the combined analysis as well as the sex-specific analyses (**b**). Sex-specific eQTLs exhibit a larger effect in one sex than the other, and the effect is diluted in the combined analysis (**c**, **d**). Sex-shared large effect eQTLs can be detected in sex-specific and combined analyses. Global *p*-values for ANOVA are shown for each eQTL type. Adjusted *p*-values based on Kruskal-Wallis tests are shown for each pairwise comparison

To further investigate the mechanisms of sex difference in HCC etiology, we used eQTL analyses to detect germline genetic effects on tumor gene expression in both the joint and sex-stratified analyses (Fig. 3a). We detected 1204, 761, and 245 eQTLs in the combined, male-specific, and female-specific analyses, respectively (Additional file 3: Tables S12–S14). As expected, genomic annotations show that most eQTLs are located on non-coding regions (Fig. 3b Additional file 3: Tables S15-S17). Consistent with previous reports, most *cis*-eQTLs were located near transcription start sites (TSSs), with 63% of all eQTLs across the combined and sex-specific analyses being located within 20 kb of TSSs. On average, 384 variants were tested per gene. 31% of the unique shared and sex-specific *cis*-eQTLs in HCC were also identified as eQTLs in the liver data in the GTEx project analysis release V7, indicating shared tissue origin. Out of the total of 1595 unique associations, 75.7% were shared between the sexes. We detected 295 male-specific and 92 female-specific eQTLs. Since these associations were not detected in the unstratified analysis, they are likely not a result of differential power to detect associations due to different sample sizes, but exhibit effect modification by sex. Sex-specific associations exhibited differences in effect size between the sexes (based on ANOVA/Kruskal-Wallis tests, Fig. 4c, d), and the sex-specific effect is diluted in the combined analysis (Fig. 4c, d). Sex-shared large effect eQTLs were detected in sex-specific and combined analyses (Fig. 4a), and, due to the larger sample size, sex-shared low-effect eQTLs are detected in the combined analysis only (Fig. 4b).

We detected 27 shared eGenes that were associated with independent variants in males and females. This could be due to actual biological differences in gene regulation, or due to technical constraints, in particular, missing genotypes in one sex affecting the permutation scheme to select the top-variant for each target gene. To overcome this and to detect high confidence instances of differential gene regulation between the sexes, we further examined the sex-shared and sex-specific eGenes: we found 24 genes that are under germline regulatory control in only male HCC (Fig. 3c), including *POGLUT1*, which is an essential regulator of Notch signaling (Fig. 3d). No genes were found to be associated with nearby variants in females only, likely due to reduced statistical power to detect associations in females (Additional file 2: Figure S2). Male-specific eGenes were overrepresented in pathways related to cell cycle, apoptosis, and cancer (Additional file 3: Table S18). Concordant with previous studies [14, 35], none of the male-specific eGenes were differentially expressed between male and female HCC, indicating that the male-specific eQTLs are not a result of differences in overall gene expression levels between males and females, but are likely to arise from factors such as differential chromatin accessibility or transcription factor activity. The observation that none of the sex-biased autosomal genes in tumors harbor significant *cis*-eQTLs (Additional file 3: Table S19) also suggests that while sex-specific *cis*-eQTLs may contribute to differences in variance, sex-biased gene expression is likely a result of *trans*-effects, e.g. sex-chromosomal effects on autosomal gene expression, or, more widely, a result of sex as a biological variable, e.g. hormonal effects.

## Discussion

### Distinct molecular etiologies of male and female HCC

It is well established that patterns of gene expression vary between the sexes across different tissues. Previous studies have confounded these differences with those which may be driving etiological differences between male and female tumors. For example, Yuan et al. previously reported extensive sex-biased signatures in gene expression in HCC and other strongly sex-biased cancers [11]. While they identified immunity and cancer-associated enriched pathways based on sex-biased genes detected in HCC tumors, their approach was limited as it did not include the examination of non-diseased liver nor tumor-adjacent tissues. From the results presented here, we are able to distinguish the differences detected in comparisons of male and female HCC from those reflecting sex differences in the healthy liver or in tumor-adjacent tissue, as well as to detect genes that are dysregulated in HCC in a sex-shared or sex-specific manner.

We characterized differences in gene expression between male and female HCC cases. Notably, sex differences in gene expression were the largest in the tumor tissue, with 53 genes (including 32 autosomal genes) being expressed in a sex-biased way. These sex differences point to distinct mechanisms underlying HCC oncogenesis between the sexes, and may partially underlie the observed sex-biases in HCC occurrence and onset. We detected 34 genes that were expressed in a sex-biased way in HCC tumors, but not in healthy or tumor-adjacent liver tissues. Some of these genes are of particular interest in the context of HCC: female-biased *CXCL14* and *ATF5* may modulate antitumor immune responses and have a tumor suppressor role in HCC [36, 37]. Additionally, *HAMP* and *GPR37* were found downregulated in male tumors in comparison to female tumors. Downregulation of *HAMP* contributes to aggressive HCC [38], and low level of *GPR37* is associated with disease progression and poor survival in HCC [39]. These genes could be considered as diagnostic biomarkers and potential targets in the treatment of male HCC. On the other hand, we detect female-biased genes that may contribute to HCC aggressiveness in females: overexpression of *FGFR2* has been associated with advanced clinical stages [40], and *NTS* is known to induce local inflammation and to promote tumor invasion in HCC [41]. Furthermore, female-biased *GGT6* has previously been identified as a potential biomarker in renal cell carcinoma [42], but has not been studied in the context of HCC. Notch-regulating *DTX1*, found here to be underexpressed in males compared to females, has been identified as a putative tumor suppressor gene in head and neck squamous cell carcinoma [43]*.* Another female-biased gene detected here, *CD24*, has a crucial role in T cell homeostasis and autoimmunity [44]. The opposing roles of *CD24* expression in cancer and autoimmune diseases raise interesting questions on the role of sex differences in immunity underlying sex differences in cancer. Future studies will focus on better understanding the differential regulation of immune functions between the sexes, and how these differences contribute to the observed biases in disease occurrence and etiology.

By sex-specific analyses of matched tumor and tumor-adjacent samples, we detected genes that are uniquely dysregulated in male and female HCC. Further examination of these genes revealed sex differences in the pathway activation, indicating that the molecular etiologies of male and female HCC are partly driven by distinct functional pathways. Males and females differed in the activation of several signaling pathways, with females showing PPAR pathway enrichment while males showed PI3K, PI3K/AKT, FGFR, EGFR, NGF, GF1R, Rap1, DAP12, and IL-2 signaling pathway enrichment (Fig. 1e, Additional file 3: Tables S9–S10). As these signaling pathways are notable targets for anti-cancer and anti-metastasis therapies [45–51], the results presented here have implications for the targeted treatment of male and female HCC.

### Sex-specific germline genetic effects on tumor gene expression may drive the molecular etiologies of male and female HCC

Sex-specific regulatory functions may underlie sex differences in cancer etiology, progression, and outcome. We detected sex differences in the germline genetic regulation of tumor gene expression in HCC, including 24 genes that were under germline regulatory control only in male HCC (Fig. 3). Functional annotations of these male-specific eGenes provide insight into possible regulatory mechanisms contributing to the observed male-bias in HCC and sex differences in HCC etiology. Protein O-glucosyltransferase 1 (*POGLUT1*) was found to be under germline regulation in male HCC, but not in female HCC nor in the joint analysis of both sexes (Fig. 3d). The eQTL associated with *POGLUT1* is located on a promoter region of its target (Additional file 3: Table S15). *POGLUT1* is an enzyme that is responsible for O-linked glycosylation of proteins. Altered glycosylation of proteins has been observed in many cancers [52, 53], including liver cancer [54, 55]. *POGLUT1* is an essential regulator of Notch signaling and is likely involved in cell fate and tissue formation during development. Genes involved in Notch and PI3K/AKT signaling were also found to be expressed in a sex-biased way in HCC tumors and overrepresented among the male-specific DEGs detected in the tumor vs. tumor-adjacent comparison, showing that sex-specific eQTLs and sex-specific dysregulated genes converge in canonical pathways. Notch signaling pathway was also detected as overrepresented (FDR-adj. *p*-value ≤0.01) among the 24 male-specific eGenes. PI3K-AKT is known to co-operate with Notch by triggering inflammation and immunosuppression [56]. Concurrent activation of Notch and PI3K/AKT pathways can trigger tumorigenesis and is prevalent in aggressive cancers [57–60]. We find simultaneous activation of PI3K/AKT and Notch pathways in male HCC, and sex-specific genetic effects on regulation of genes involved in PI3K/AKT signaling. These results point to a major role of the Notch/PI3K/AKT axis in the development of HCC in males. PI3K/AKT signaling is of particular interest in the context of HCC, as it has been implicated in HCC carcinogenesis [61], is involved in hepatic gluconeogenesis [62], and is activated in a sex-biased way in the liver and other tissues [63]. The role of Notch and PI3K/AKT signaling in HCC may differ between early and late-stage tumors and among molecular subtypes, and further studies are necessary to understand the oncogenic properties of these pathways among HCC subtypes and between the sexes. In the future, analyses of data collected as a part of the International Cancer Genomics Consortium project may elucidate the sex-specific processes of HCC oncogenesis among the Japanese, as well as the interactions between sex and hepatitis infections in shaping HCC etiology. However, each dataset has a unique ancestry composition and are not directly comparable for validation purposes.

## Conclusions

In summary, we discovered differential regulatory functions in HCC tumors between the sexes. This work provides a framework for future studies on sex-biased cancers. Further studies are required to identify and validate sex-specific genetic effects on tumor gene expression and its consequences in HCC and other sex-biased cancers across diverse populations.

## Supplementary information

**Additional file 1: Figure S1.** A multi-dimensional scaling plot of the TCGA LIHC tumor samples of each sex (N male = 248, N female = 119). Euclidean distances between samples were calculated based on 100 genes with the largest standard deviations between samples.

**Additional file 2: Figure S2.** Estimation of statistical power in the combined (grey), male-specific (blue), and female-specific (red) eQTL analyses with a *p*-value level 0.01 and 384 variants. Increased power in the combined analysis allows the detection of sex-shared low-effect eQTLs.

**Additional file 3: Tables S1-S19. **

## Data Availability

Data used in this study are available at dbGaP at https://www.ncbi.nlm.nih.gov/gap/ and NCI Genomic Data Commons at https://gdc.cancer.gov/.

## Abbreviations

DEG: Differentially expressed gene
eQTL: Expression quantitative trait loci
HBV: Hepatitis B virus
HCC: Hepatocellular Carcinoma
HCV: Hepatitis C virus
TSS: Transcription start site
