# Supplementary figures and images for: Distinct molecular etiologies of male and female hepatocellular carcinoma
Source: BMC Cancer. 2019 Oct 15;19:951. doi: 10.1186/s12885-019-6167-2 (PMC6794913; doi:10.1186/s12885-019-6167-2)

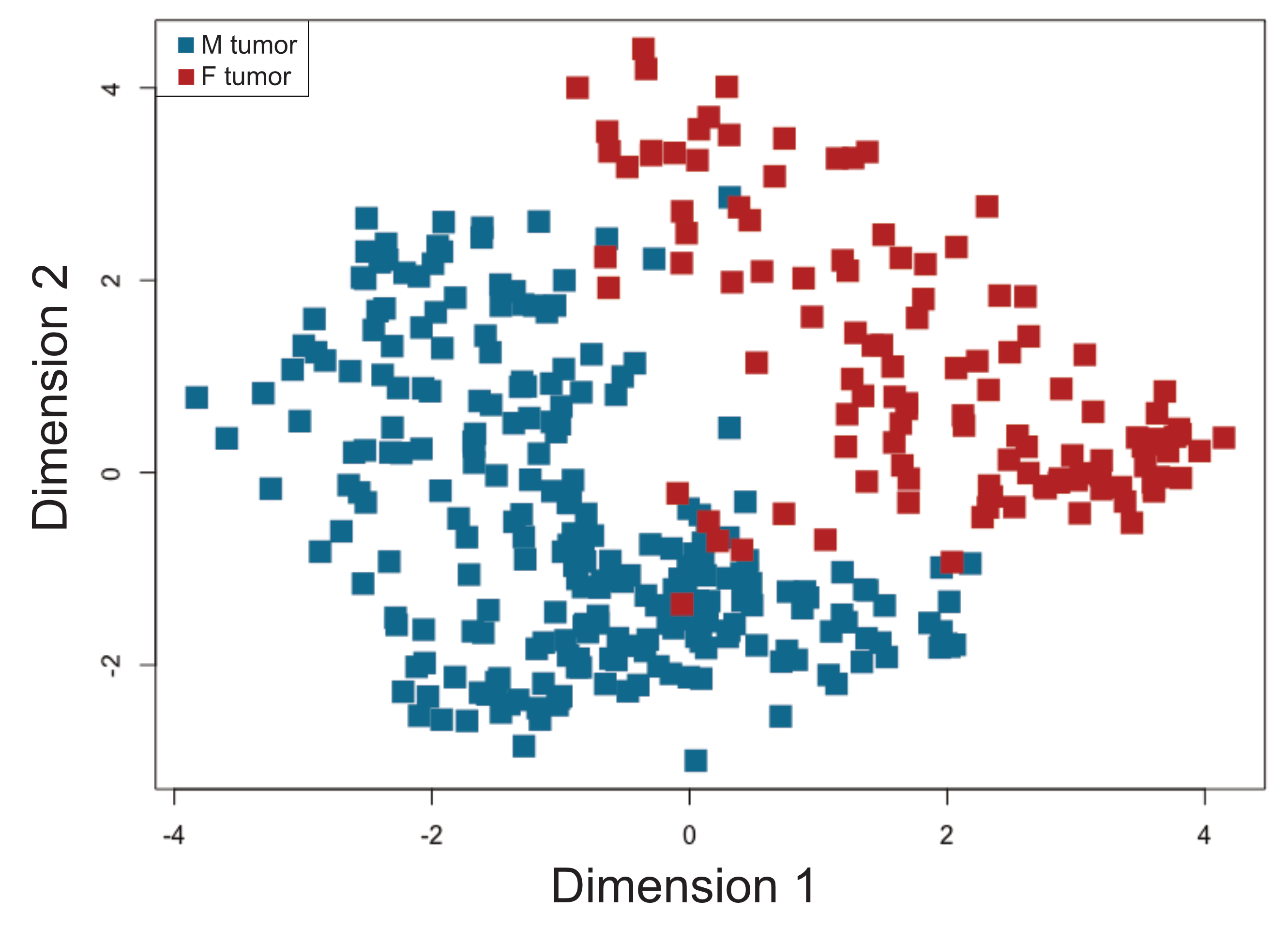

Supplement: Supplementary file 1 — Additional file 1: Figure S1. A multi-dimensional scaling plot of the TCGA LIHC tumor samples of each sex (N male = 248, N female = 119). Euclidean distances between samples were calculated based on 100 genes with the largest standard deviations between samples. [file 12885_2019_6167_MOESM1_ESM.png]

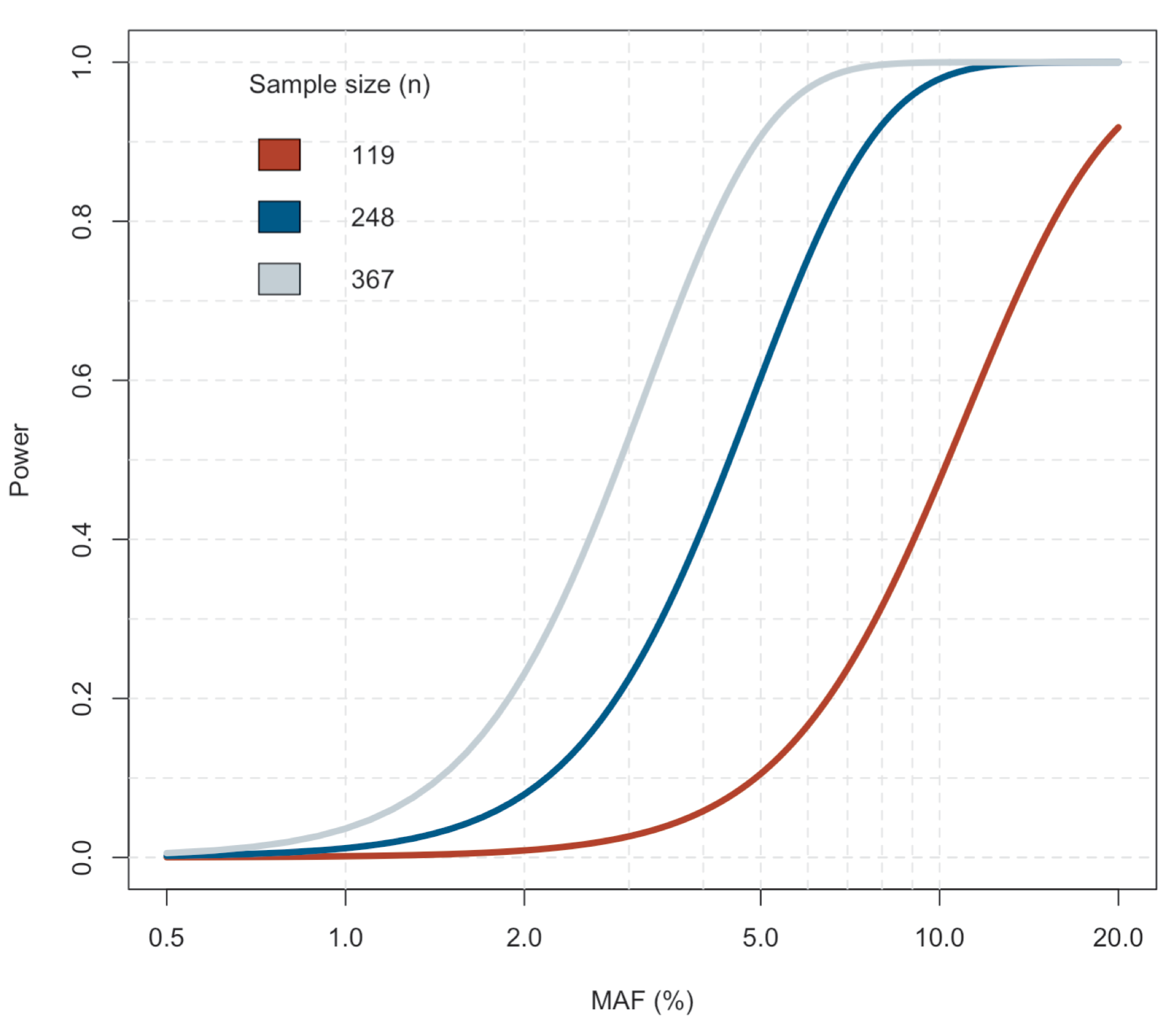

Supplement: Supplementary file 2 — Additional file 2: Figure S2. Estimation of statistical power in the combined (grey), male-specific (blue), and female-specific (red) eQTL analyses with a p-value level 0.01 and 384 variants. Increased power in the combined analysis allows the detection of sex-shared low-effect eQTLs. [file 12885_2019_6167_MOESM2_ESM.png]
